# Physiologic Functions and Therapeutic Applications of α7 Nicotinic Acetylcholine Receptor in Brain Disorders

**DOI:** 10.3390/pharmaceutics15010031

**Published:** 2022-12-22

**Authors:** Chien-Hsing Lee, Shih-Ya Hung

**Affiliations:** 1 Department of Pharmacology, School of Post-Baccalaureate Medicine, College of Medicine, Kaohsiung Medical University, Kaohsiung 80708, Taiwan; 2 Department of Medical Research, Kaohsiung Medical University, Chung-Ho Memorial Hospital, Kaohsiung 80708, Taiwan; 3 Drug Development and Value Creation Research Center, Kaohsiung Medical University, Kaohsiung 80708, Taiwan; 4 Department of Biological Science and Technology, National Pingtung University of Science and Technology, Pingtung 91201, Taiwan; 5 Graduate Institute of Acupuncture Science, China Medical University, Taichung 40402, Taiwan; 6 Division of Surgery, Department of Medical Research, China Medical University Hospital, Taichung 40447, Taiwan

**Keywords:** α7nAChR, Alzheimer’s disease, depression, Parkinson’s disease, schizophrenia

## Abstract

Accumulating data suggest that α7 nicotinic acetylcholine receptors (α7nAChRs) are an important therapeutic target for the treatment of Alzheimer’s disease (AD) and schizophrenia. The homopentameric ligand-gated ion channel α7nAChR consists of five identical α7 subunits that are encoded by the *CHRNA7* (cholinergic receptor nicotinic alpha7 subunit) gene. Moreover, α7nAChRs are densely distributed throughout the hippocampus, cortex, and thalamus brain regions, but sparsely in the striatum, forebrain, and medulla. Compared with other nAChRs, α7nAChR binds with low affinity to the naturally occurring neurotransmitter acetylcholine and the non-specific exogenous agonist nicotine, and with high affinity to the specific antagonists α-bungarotoxin and methyllycaconitine. Reports indicate that α7nAChR plays important roles in neurotransmitter release, cognitive functioning, and the cholinergic anti-inflammatory response. Genetic variations that alter *CHRNA7* mRNA and protein expression or cause α7nAChR dysfunction are associated with many brain disorders. Our previous studies revealed that α7nAChR exerts neuroprotection in AD by acting as a cargo receptor for binding the autophagosomal marker protein LC3 and engulfing extracellular neurotoxic Aβ_1–42_ during autophagic degradation of the α7nAChR-Aβ_1–42_ complex. However, the role of α7nAChRs in other diseases remains unknown. Here, we review and summarize the essential characteristics and current findings concerning α7nAChRs in four common brain diseases (AD, Parkinson’s disease, schizophrenia, and depression), which may elucidate the role of α7nAChRs and inform innovative research and novel treatments that target α7nAChRs in brain disease.

## 1. Introduction of Cholinergic Receptors and Nicotinic Acetylcholine Receptors (nAChRs)

**Figure 1:**
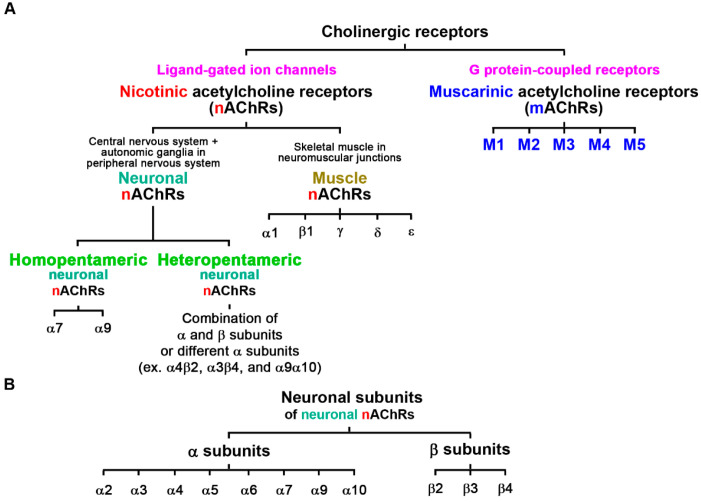
Classification of cholinergic receptors and subunits of neuronal nicotinic acetylcholine receptors (nAChRs). (**A**) Cholinergic receptors contain nicotinic and muscarinic acetylcholine receptors. (**B**) Neuronal subunits of neuronal nAChRs in mammalians (α8 is cloned from the chicken brain).

The cholinergic system is a major excitatory pathway that modulates the activity of the nervous system. Acetylcholine is a naturally occurring neurotransmitter found in the brain, neuromuscular junctions, and autonomic ganglia. Cholinergic receptors are stimulated by endogenous acetylcholine and enable signal transduction in the nervous system. Cholinergic receptors are subdivided into active nicotinic acetylcholine receptors (nAChRs) and muscarinic acetylcholine receptors (mAChRs), which belong to the exogenous agonists nicotine and muscarine, respectively (Figure 1A) [1]. The mAChRs are a subfamily of G protein-coupled receptors that use G proteins as their signaling mechanism; mAChRs contain M1, M2, M3, M4, and M5 subtypes that modulate the activity of a huge number of physiological functions in the central and peripheral nervous systems (Figure 1A) [2]. M1, M3, and M5 subtypes are coupled to G_q_ proteins that activate phospholipase C for stimulation-type effects; M2 and M4 subtypes are coupled to G_i/o_ proteins that repress adenylate cyclase activity for inhibitory-type effects [2]. Cell-surface nAChRs are evolutionarily conserved receptors that are widely distributed throughout the animal kingdom, from nematodes to humans [3]. nAChRs are ligand-gated ion channels activated by the endogenous agonist acetylcholine and exogenous agonist nicotine, triggering increases in levels of intracellular cations, including Ca^2+^ [4]. In vertebrate and non-vertebrate animals, nAChRs are broadly categorized into neuronal and muscle nicotinic acetylcholine receptors based on their primary sites of expression (Figure 1A). Neuronal nAChRs exist on neurons of the autonomic ganglia in the peripheral nervous system and many areas of the central nervous system (CNS); muscle nAChRs exist on cells of skeletal muscle in the neuromuscular junction, which is the target of muscle relaxants [5]. Different neuronal nAChRs consist of five neuronal subunits that form a central ion pore. In mammals, neuronal subunits of nAChRs are divided into α subunits (α2, α3, α4, α5, α6, α7, α9, and α10; α8 is cloned from the chicken brain) and β subunits (β2, β3, and β4) (Figure 1B) [6]. Homopentameric neuronal nAChRs are formed by five identical α7 or α9 subunits; heteropentameric neuronal nAChRs combine α and β subunits or different α subunits, such as α4β2, α3β4, and α9α10 (Figure 1B). The most commonly found neuronal nAChRs in the mammalian brain are the homopentameric α7nAChRs and the heteropentameric α4β2nAChRs [6]. For receptor activation, heteropentameric nAChRs containing both α and β subunits bind nicotine with high affinity (nM) and homopentameric α7nAChR bind nicotine with low affinity (μM); α4β2nAChRs contribute >90% of the high-affinity binding sites for nicotine in the rat brain [7]. Human genes that encode for each nAChR neuronal subunit are listed in Table 1, including the reference sequences of proteins, genomic DNA, and mRNA transcripts, genomic DNA locations, and protein and mRNA lengths.

## 2. α7 Nicotinic Acetylcholine Receptor (α7nAChR)

The homopentameric α7nAChR is unique among nAChRs, because of its high Ca^2+^ permeability, relatively low sensitivity to acetylcholine (EC_50_ = 30 μM), full activation by choline as a full agonist (EC_50_ = 0.4–1.6 mM), high-affinity for α-bungarotoxin (from the snake *Bungarus multicinctus*), relatively low affinity for nicotine (EC_50_ = 18–91 μM), and fast desensitization as determined by the submillisecond time scale following exposure to agonists [8]. In the brain, high levels of α7nAChR are found in regions implicated in cognitive function and memory, including the hypothalamus, geniculate nuclei, colliculi, hippocampus, medial habenula, thalamus, cortex, and amygdala; levels of α7nAChR are low in the striatum, forebrain, medulla and various brain nuclei [8,9]. α7nAChRs are expressed in presynaptic and postsynaptic locations that facilitate neurotransmitter release and in perisynaptic locations that activate signaling pathways through volume transmission [8]. Presynaptic and postsynaptic α7nAChRs modulate neurotransmitter release in the brain through Ca^2+^-dependent mechanisms, and α7nAChRs regulate neuronal growth and differentiation in the developing CNS [7]. In non-neuronal cells, including astrocytes, microglia, dendritic cells, lymphocytes, macrophages, and endothelial cells, α7nAChRs play a role in immunity, inflammation, and neuroprotection [8]. Moreover, α7nAChR binds the non-specific exogenous agonist nicotine with low affinity and the specific exogenous antagonists α-bungarotoxin (IC_50_ = 1–100 nM) and methyllycaconitine (IC_50_ = 10–200 nM) with high affinity [8,10]. Surface-expressed α7nAChRs in cells can be detected by [^125^I]-α-bungarotoxin labeled with ^125^I on tyrosine residue 54 measured by radioligand binding or sucrose gradient centrifugation [11]. PC12 (rat pheochromocytoma) and SH-SY5Y (human neuroblastoma) cells express endogenous α7nAChR on the cell surface; the dissociation constant (K_D_) value of [^125^I]-α-bungarotoxin binding to endogenous α7nAChRs in untransfected SH-SY5Y cells is 0.6 ± 0.3 nM [11].

The cell-surface homopentameric α7nAChRs facilitate the flux of Na^+^ and K^+^ and have high permeability for Ca^2+^ after stimulation by agonists [8,12]. According to *Homo sapiens CHRNA7* (cholinergic receptor nicotinic alpha7 subunit) data from the NCBI (National Center for Biotechnology Information; Gene ID: 1139; https://www.ncbi.nlm.nih.gov/gene/1139 (accessed on 5 December 2022)), the α7 subunit structure is believed to consist of a conserved N-terminal extracellular domain followed by three conserved transmembrane domains, a variable cytoplasmic loop, a fourth conserved transmembrane domain, and a short C-terminal extracellular region. Dysfunction of the α7 subunit is associated with several neurologic and neuropsychiatric disorders, such as Alzheimer’s disease (AD) and schizophrenia [8]. The expression of α7nAChRs in the immune system is of particular interest, as this system plays a crucial role in regulating the cholinergic anti-inflammatory pathway [13]. Nicotinic neuroprotection by α7nAChRs modulates neuroinflammation through increased calcium permeability [14]. Thus, α7nAChR potentiation has emerged as a therapeutic strategy in progressive neurologic disorders.

## 3. The *CHRNA7* Gene

According to *Homo sapiens CHRNA7* data from the NCBI, human *CHRNA7* DNA contains ten exons and spans approximately 75 kb on chromosome 15q13.3 (NCBI reference sequence: NC_000015.10) [15,16,17] (Table 1). Exons one to six of *CHRNA7* correspond to the receptor’s extracellular N-terminal region, which contains the ligand-binding domain; exons seven and eight correspond to the first three transmembrane regions, M1, M2, and M3, with M2 constituting the ion channel of the receptor; exons nine and ten encode its intracellular cytoplasmic loop, the fourth transmembrane region, M4, and the extracellular C terminus [18]. Next-generation sequencing (NGS) data of RNA sequencing reveal the presence and quantity of *CHRNA7* RNA in 27 human tissues and show that the top eight tissues that express *CHNRA7* mRNA are the adrenal, small intestine, testis, stomach, duodenum, thyroid, colon, and brain [19]. *CHRNA7* mRNA expression has been detected as early as embryonic Day-13 (E13) in the murine peripheral nervous system (including dorsal root ganglia, parasympathetic and sympathetic ganglia), with the strongest expression in the superior cervical ganglia, and low to moderate levels in the brain and spinal cord, respectively; the intensity of mRNA expression rapidly increased with embryonic age [12]. The autoradiographic images also revealed that *CHRNA7* mRNA was expressed throughout the CNS, including the spinal cord and retina, as early as E13 and up until birth [12]. These data indicate that *CHRNA7* mRNA is abundantly expressed in the CNS, peripheral nervous system, and spinal cord.

## 4. Transcriptional Factors, Promoter DNA Methylations, Tobacco Smoking, and Promoter Variants Regulate Human *CHRNA7* mRNA Expression

Alterations in *CHRNA7* mRNA and protein expression are associated with many diseases, such as AD, Parkinson’s disease (PD), schizophrenia, autism, and microdeletion syndromes [20]. Human *CHRNA7* mRNA transcription is regulated by activating protein 2α (AP-2α)-mediated transcriptional repression, promoter DNA methylation, promoter variants (mutations or polymorphisms) in the proximal region, and tobacco smoking [16,17,21,22]. The human proximal *CHRNA7* promoter (−245 to −1 of the translation start site) contains consensus binding sequences of transcription factors, including multiple Sp (specificity protein) and Egr-1 (early growth response 1) binding sites, and a single AP-2α (activator protein-2α) binding site [21]. One study has demonstrated that AP-2α specifically binds to the *CHRNA7* promoter at the 71-base (immediately upstream of the translation start site) and thus represses *CHRNA7* mRNA expression in SH-SY5Y cells [21]. The DNA sequence of the *CHRNA7* promoter region is GC-rich and has no canonical CCAAT- or TATA-box, the proximal promoter region of *CHRNA7* promoter is highly methylated in a non-neuronal epithelial cell line SH-EP1 derived from the SH-SY5Y cell line [21]. AP-2α is a potent transcriptional repressor of the *CHRNA7* gene; DNA methylation in the proximal promoter region reduces *CHRNA7* mRNA expression in human cells [21]. In analyses of the postmortem hippocampus, *CHRNA7* mRNA and protein levels are normal in schizophrenic smokers compared with control non-smokers, whereas schizophrenic non-smokers have significantly lower levels of *CHRNA7* mRNA and protein expression compared with schizophrenic smokers [17]. The reduction in hippocampal *CHRNA7* mRNA in schizophrenic non-smokers is associated with a greater proportion of promoter polymorphisms (−86 C/T, −194 G/C, and −46 G/T upstream from the transcriptional start site) compared with control non-smokers and schizophrenic smokers [17]. It is speculated that promoter polymorphism accounts for the low levels of *CHRNA7* mRNA in schizophrenic non-smokers [17]. Other research has identified six of eight promoter variants (mutations or polymorphisms) in the proximal region (the 231-bp core promoter region directly upstream of the transcriptional start site) of the *CHRNA7* gene reduce transcription as measured by the luciferase reporter gene assay in SH-SY5Y cells [22]. These data suggest that human *CHRNA7* mRNA expression is regulated by the AP-2α transcriptional factor, promoter DNA methylation, tobacco smoking, and promoter variants (mutations or polymorphisms).

## 5. Aberrant α7 Subunit Trafficking, Folding, and Assembly Reduces Cell-Surface Expression of Functional α7nACRs

The assembly of AChR is a slow and inefficient process, with only 30% of newly synthesized subunits forming functional receptors after adopting the correct transmembrane topology and undergoing critical post-translational modifications [23]. The formation of functionally expressing α7nAChRs at the cell surface is believed to involve a tightly regulated process whereby α7 subunits undergo post-translational modifications, assembly by subunit-subunit interactions, and cell-surface delivery. However, the precise mechanisms of α7 subunit folding and α7nAChR assembly are unclear. Cooper and Millar (1997) found that the five α7nAChR receptor subunits undergo folding and assembly as homopentamers in the endoplasmic reticulum and migrate through the Golgi to the cell surface [11]. Immunofluorescent labeling of the α7 subunit in *CHRNA7*-transfected human embryonic kidney 293 (HEK293) cells has revealed that misfolded α7 is aggregated and retained in the endoplasmic reticulum [11]. The assembly of α7 subunits into homopentameric receptors and α7nAChR transportation to the cell surface are host-cell-specific [11]. Human RIC3 (resistant to inhibitors of cholinesterase 3) mRNA is present in neuronal SH-SY5Y cells and absent in non-neuronal HEK293 cells [24]. Moreover, cell-surface α7nAChRs are recognized by α-bungarotoxin only in mammalian cells expressing RIC3, and RIC3 co-expression with α7nAChRs reportedly increases the magnitude of acetylcholine-induced currents in *Xenopus* oocytes [24]. Co-expression of *CHRNA7* and *RIC3* cDNAs promotes the formation of functional α7nAChRs on the surface of non-neuronal mammalian HEK293 cells [24], while NACHO (a transmembrane protein of neuronal endoplasmic reticulum) mediates α7nAChR assembly and synergizes with RIC3 [25]. Furthermore, NACHO promotes α7 subunit folding, maturation through the Golgi, and membrane insertion for α7nAChR expression at the cell surface [25]. Electrophysiologic recordings of hippocampal neurons have shown a complete absence of α7nAChR-mediated acetylcholine-evoked currents in NACHO knockout rats, suggesting that NACHO is required for α7nAChR functioning in the brain [25]. The endogenous prototoxin lynx1 is highly expressed in the mammalian CNS; applying soluble lynx1 to α7nAChRs increases acetylcholine-evoked macroscopic currents in *Xenopus* oocytes [26]. Lynx1 binds directly to α7nAChRs in mammalian HEK293T cells [27]. The [^125^I]-α-bungarotoxin binding measurement of α7nAChR cell-surface expression has revealed an approximate 50% decrease in the postmortem hippocampus of schizophrenic subjects compared with hippocampus from non-schizophrenic subjects [28]. It has been suggested that the hippocampus of schizophrenic smokers contains adequate α7 protein but low α-bungarotoxin binding, which may interfere with the assembly or trafficking of α7nAChR [16]. These data demonstrate that α7 subunit folding or α7nAChRs assembly into a functional receptor on the cell surface depends on the host cell and the presence of proteins such as RIC3 and lynx1, while α7 subunit trafficking, folding, and assembly affects cell-surface α7nAChR expression.

## 6. Co-Assembly of the Dupα7 and α7 Subunits Impairs α7nAChR Functions

*CHRFAM7A* is a protein-coding gene and human-specific chimeric gene, with incompletely characterized patterns of expression and functions [16,29]. The genomic order of the upstream exons of *CHRFAM7A* gene contains exon D of unknown provenance, exons C, B, and A duplicated from the *ULK* (*4unc-51 like kinase 4*) gene, and exons five, six, seven, eight, nine and ten duplicated from the *CHRNA7* gene [16]. *CHRFAM7A* is located at 15q13-14, almost always in the opposite orientation to *CHRNA7* [18]. While *CHRFAM7A* is transcribed efficiently, it is poorly translated [30]. The protein product of *CHRFAM7A* is the dupα7 subunit, a partially duplicated isoform of the human α7 subunit [30]. The dupα7 subunit lacks the signal peptide and a ligand-binding domain on the extracellular N-terminal region, but contains all of the α7 membrane-spanning regions [18]. Each dupα7 subunit co-assembles with four α7 subunits to form a functional heteropentameric α7dupα7 receptor in mouse neuroblastoma Neuro2a cells [18], which removes two of the five agonist binding sites [30]. In *Xenopus* oocytes, *CHRNA7* and *CHRFAM7A* co-expression does not alter transcription from either gene, but causes a reduction in the average acetylcholine-evoked current and [^125^I]-α-bungarotoxin binding site at the cell surface [16]. The incorporation of dupα7 and dupΔα7 (exon six of *CHRFAM7A* harbors a 2-bp deletion polymorphism) subunits modestly changes the sensitivity of receptors to choline and varenicline in mouse neuroblastoma Neuro2a cells [18], while the stable overexpression of dupα7 inhibits α7nAChR-induced intracellular concentration of Ca^2+^ signaling and exocytotic responses in human SH-SY5Y cells [31]. Using small interfering RNAs (siRNAs) to silence *CHRFAM7A* expression enhances α7nAChR-induced dopamine release in SH-SY5Y cells [31]. These data indicate that the dupα7 and α7 subunit combination is a dominant negative regulator of α7nAChR functions capable of reducing α7nAChR-mediated acetylcholine-induced currents, intracellular concentrations of Ca^2+^ signaling, and dopamine release.

## 7. α7nAChR in AD and Therapeutic Applications

AD is the most common neurodegenerative disease worldwide and is characterized by progressive memory loss and cognitive impairment [32]. In 2022, an estimated 6.5 million Americans aged 65 years and older were living with AD, which was predicted to increase to 13.8 million by 2060 [33]. The five neuropathologic hallmarks found in the brains of AD patients are acetylcholine deficiency, glutamate excitotoxicity, extracellular deposition of amyloid-β (Aβ) plaques, intraneuronal neurofibrillary tangles (NFTs) with hyperphosphorylated tau, and neuroinflammation [32]. These markers have been targeted by clinical trials in drug development research exploring the utility of acetylcholinesterase inhibitors, agonists and antagonists of neurotransmitter receptors, β-secretase (BACE) and γ-secretase inhibitors, vaccines and antibodies targeting Aβ clearance or tau protein, and anti-inflammatory compounds [32]. Loss of cholinergic tone and acetylcholine levels in the brain are thought to be responsible for the gradual cognitive decline in AD patients [34]. The cholinesterase inhibitors donepezil, galantamine, and rivastigmine are first-line treatment options used to enhance brain acetylcholine levels in AD patients [32].

Analyses conducted in 1981 of postmortem brain tissue samples from patients with AD reported reductions in [^125^I]-α-bungarotoxin binding sites of 22.3% in the frontal cortex and 39.2% in the mid-temporal gyrus [35]. Other researchers have reported finding a 25% reduction in [^125^I]-α-bungarotoxin binding in the hippocampus of AD patients compared with age-matched controls; binding sites for [^125^I]-α-bungarotoxin in the temporal cortex of AD brain did not differ from controls and were significantly increased by 39% in the cerebellum of AD brains [36]. Guan et al. (2000) reported a 36% reduction in α7 subunit protein expression in hippocampal samples from AD patients compared with age-matched controls, with no significant differences in temporal cortex expression between AD and control brains [37]. Interestingly, Hellstrom-Lindahl et al. (1999) found that *CHRNA7* mRNA levels were increased by 65% in the hippocampus of AD brains compared with control samples [36]. This suggests that the increase in *CHRNA7* mRNA may occur as a compensatory mechanism to maintain α7nAChR function in the AD brain. Since α7nAChR plays an important role in learning and memory and is found in key brain regions associated with AD, including the cerebral cortex and hippocampus, compounds are designed to target α7nAChR as a potential therapeutic strategy for AD.

Anabaseine (3,4,5,6-tetrahydro-2,3′-bipyridine) is a marine invertebrate (worm) toxin and is structurally related to nicotine; the anabaseine analog 3-(2,4-dimethoxybenzylidene)-anabaseine dihydrochloride (DMBX-anabaseine, DMXB, DMXB-A, or GTS-21) is a selective agonist of α7nAChR. Qi et al. (2007) found that GTS-21 exerts neuroprotection by reducing Aβ_25–35_-induced neuronal death and lipid peroxidation in SH-SY5Y cells, while siRNA-induced silencing of the α7 subunit enhanced these toxic effects [38]. The preclinical profile of GTS-21 led to its development for the treatment of both cognitive dysfunction and neurodegeneration seen in AD patients. In four phase I trials, a total of 87 healthy volunteers were enrolled to evaluate GTS-21; the data indicate GTS-21 was well tolerated up to doses of 450 mg/day (150 mg three times a day) and superior to placebo regarding cognitive function and working memory in normal subjects [39]. Encenicline (EVP-6124 or MT-4666, FORUM Pharmaceuticals Inc., Watertown, MA, USA and Mitsubishi Tanabe Pharma, Osaka, Japan) is a selective partial agonist of α7nAChRs (Ki = 4.3 nM) and acts as a co-agonist with acetylcholine to enhance cognition; encenicline was in phase III clinical trials for AD treatment but was discontinued in 2015 due to gastrointestinal side effects [32]. Extracellular deposition of Aβ plaques is a neuropathological hallmark of AD, and Aβ_1–42_ is the major component of Aβ plaques [32]. Nagele et al. (2002) found that Aβ_1–42_ accumulates in neurons and Aβ plaques in postmortem brain tissues of the entorhinal cortex and hippocampus from patients with sporadic AD, but not in age-matched controls [40]. Aducanumab (marketed as Aduhelm; Biogen, Cambridge, MA, USA) is an anti-Aβ monoclonal antibody that reduces brain Aβ plaques in AD patients by enhancing Aβ clearance through passive immunotherapy [32]. In June 2021, the United States Food and Drug Administration (US FDA) approved Aduhelm under the accelerated approval pathway, requiring Biogen to verify the clinical benefit of Aduhelm in a post-approval trial [41]. If Biogen cannot verify the clinical benefit of Aduhelm, the US FDA may initiate proceedings to withdraw its approval of Aduhelm [41]. Biogen filed the final design for the Aduhelm post-approval study to the US FDA in March 2022.

Three studies have demonstrated that extracellular Aβ_1–42_ has a higher affinity than Aβ_1–40_ for binding to α7nAChR, and that α7nAChR further facilitates Aβ_1–42_ entry and intraneuronal accumulation via endocytosis [40,42,43]. Nagele et al. (2002) found that α7nAChR overexpression in human neuroblastoma SK-N-MC cells transfected with *CHRNA7* was characterized by rapid binding, internalization, and accumulation of exogenous Aβ_1–42_ (but not Aβ_1–40_) via endocytosis [40]. Immunohistochemical evidence has shown that α7nAChR is present in AD neuritic plaques and co-localizes with Aβ_1–42_ in individual cortical neurons [42]. Researchers have also found that α7nAChR and Aβ_1–42_ form a stable complex in hippocampal membrane proteins prepared from patients with sporadic AD and age-matched nondemented controls [42]. An investigation using the α7nAChR selective radioligand [^3^H]methyllycaconitine has demonstrated a very high-affinity reaction between the binding of Aβ_1–42_ to α7nAChRs in rat hippocampal/cortical and guinea hippocampal membranes, with Ki (inhibition constant) values of 4.1 and 5.0 picomolar (pM), respectively [43]. Notably, Wang et al. (2000) demonstrated that the affinity of Aβ_1–42_ for α4β2nAChRs was approximately 100–5000-fold lower than for α7nAChRs [43]. It appears that it may be possible to prevent and reverse the binding of Aβ_1–42_ to α7nAChR, according to preclinical evidence involving the small molecule drug simufilam (PTI-125, Cassava Sciences, Austin, TX, USA), which binds to filamin to stabilize the Aβ_1–42_ and α7nAChR interaction; filamin reportedly triggers tau phosphorylation and synaptic dysfunction [44]. Following promising outcomes from phase II trials in patients with mild-to-moderate AD treated with simulfilam, two phase III trials of simuflam began in November 2021, although shortly afterwards Cassava Sciences was challenged by reports of data manipulation in several published studies and clinical trials relating to simufilam [44]. In July 2022, the United States Department of Justice opened a criminal investigation into whether Cassava Sciences manipulated research results; the results are not yet available [44]. Uncertainty surrounds simufilam for now. What is known is that Aβ_1–42_ and α7nAChR appear within AD neuritic plaques, and that Aβ_1–42_ selectively binds with high affinity to α7nAChR to form α7nAChR-Aβ_1–42_ complexes, but reductions in the α7nAChR and Aβ_1–42_ interaction may not effectively halt the disease progression in AD.

Autophagy is a cellular degradation pathway for damaged organelles and protein aggregates via lysosomal digestion; large numbers of autophagic vacuoles accumulate in the brains of AD patients [45]. In 2009, we found that extracellular Aβ_1–42_ induces a strong autophagic response [46]. In that study, we used an α7nAChR siRNA to knockdown α7nAChR expression and an Atg7 siRNA to block the autophagic process, to demonstrate that α7nAChR may act as a carrier that binds with extracellular Aβ_1–42_ and internalizes into the cytoplasm, inhibiting Aβ_1–42_-induced neurotoxicity via an autophagic degradation pathway [46]. Our data suggest that autophagy plays a neuroprotective role against Aβ_1–42_-induced neurotoxicity, and that defects in autophagic regulation or the Aβ_1–42_-α7nAChR transport system may impair the clearance of Aβ_1–42_ and enhance neuronal death [46]. LC3 is an autophagosomal marker protein that is necessary for autophagosomal membrane formation [47]. In 2015, we overexpressed enhanced green fluorescent protein-fused LC3 in both neuroblastoma cells (SH-SY5Y/pEGFP-LC3) and mice (TgEGFP-LC3) to assess the effect of LC3 overexpression on Aβ_1–42_ neurotoxicity [48]. We demonstrated that extracellular Aβ_1–42_ binding with α7nAChR is an important step in Aβ_1–42_ detoxification and that LC3 overexpression exerts neuroprotection by increasing α7nAChR expression for extracellular Aβ_1–42_ binding and further enhancing autophagic activity for Aβ_1–42_ clearance in neurons and mice [48].

Acetylcholinesterase inhibitors are the first-line agents for the treatment of AD. Galantamine is a reversible and competitive acetylcholinesterase inhibitor and may act as a positive allosteric modulator/potentiating ligand [49]. When galantamine and acetylcholine bind to their respective binding sites on pre- and post-synaptic nAChRs of cholinergic neurons, galantamine facilitates acetylcholine-induced responses [50]. Unlike galantamine, the acetylcholinesterase inhibitors rivastigmine and donepezil do not potentiate nAChR-mediated responses; donepezil is a reasonably potent inhibitor of nicotine- and KCl-evoked increases in Ca^2+^ [51]. Clinical trial data show that galantamine maintains patients’ cognitive functioning for 12 months and suggests that galantamine may have additional benefits for AD patients compared with other acetylcholinesterase inhibitors [50]. In 2020, our study found that galantamine inhibits Aβ_1–42_-induced neurotoxicity by activating MAPK/JNK signaling to enhance α7nAChR expression in SH-SY5Y cells [52]. It is also established that α7nAChR acts as a cargo receptor (binding with cargo for autophagic degradation) for autophagosomal marker protein LC3 binding and Aβ_1–42_ engulfment during the autophagic degradation of α7nAChR-Aβ_1–42_ complexes [52]. Overall, α7nAChR expression and functioning offer neuroprotection against neurotoxic Aβ_1–42_ through autophagic degradation in vitro and in vivo, and using drugs to stimulate the function or expression of α7nAChR is a potential AD therapeutic strategy [32,46,48,52].

## 8. α7nAChR in PD and Therapeutic Applications

PD is the second most common neurodegenerative disorder after AD and the most common movement disorder [53]. In 2016, approximately 6.1 million patients globally were estimated to have PD, and the age-standardized prevalence rate increased by 21.7% between 1990 and 2016 [54]. No treatment can cure PD, but treatments are available to improve PD-related symptoms and maintain patients’ quality of life [55,56]. Progressive loss of dopaminergic neurons in the substantia nigra pars compacta of the midbrain is one of the pathologic hallmarks of PD; dopaminergic neuronal death caused by unknown reasons ultimately results in severe striatal dopamine deficiency and the development of primary motor symptoms, including resting tremor, bradykinesia, muscle rigidity, and postural instability [56,57]. The nigrostriatal dopamine depletion results in multifaceted alterations in functions of the corticobasal ganglia–thalamocortical loop circuits [58]. In PD, dopamine depletion blocks the autoinhibition of acetylcholine release through muscarinic autoreceptors, leading to excessive acetylcholine release, which eventually prunes spines of the indirect-pathway projection neurons of the striatum and thus interrupts information transfer from motor command centers in the cerebral cortex [58]. Other pathogenesis features in PD include α-synuclein misfolding and aggregation, impaired functioning of mitochondria, dysregulation of protein clearance control, accumulation of oxidative stress, and neuroinflammation [55,56,57]. In addition to motor symptoms, many PD patients may also experience cognitive and behavioral problems due to anxiety, depression, and apathy [59].

The dopamine precursor and naturally occurring amino acid L-dopa (also known as levodopa) remain the gold standard for the symptomatic treatment of motor symptoms in PD [60]. Levodopa, coupled with the peripheral decarboxylase inhibitor carbidopa to facilitate levodopa availability in the brain, usually provides good control of PD motor symptoms; however, fluctuations and dyskinesias are the two main motor complications associated with chronic levodopa therapy [61]. Within five years, up to half of PD patients taking levodopa develop motor fluctuations, dyskinesias, or both [62,63,64]. One clinical study has suggested that levodopa either slows the progression of PD or has a prolonged effect on the symptoms of the disease, although the neuroimaging data indicate that levodopa may accelerate the loss of nigrostriatal dopamine nerve terminals or that its pharmacologic effects modify the dopamine transporter [65]. Thus, despite levodopa being the gold standard drug therapy for PD, these data indicate levodopa therapy-induced dyskinesia and OFF symptoms remain unresolved, and the possibility that levodopa is toxic to dopamine neurons in PD patients. It is therefore crucial that new PD neuroprotective drugs are discovered that effectively reduce levodopa-induced neurotoxicity and dyskinesias.

α7nAChR agonists exert neuroprotection against dopaminergic neuronal death and reduce levodopa-induced dyskinesias in PD animal models [9]. ABT-107 is a high-affinity and selective α7nAChR agonist that reduces PD motor deficits and nigrostriatal damage by enhancing striatal dopaminergic function in 6-hydroxydopamine-induced PD rat models [66]. GTS-21 is a selective α7nAChR agonist, while methyllycaconitine is a selective α7nAChR antagonist. In 6-hydroxydopamine-induced PD rat models, GTS-21 attenuates methamphetamine-stimulated rotational behavior, dopaminergic neurodegeneration, and glial activation; these effects are abolished by methyllycaconitine [67]. PNU-282987 is a selective α7nACh agonist; in MPTP (1-methyl-4-phenyl-1,2,3,6-tetrahydropyridine)-induced PD mouse models, PNU-28287 administration reduces neuroinflammation and dopaminergic neuronal death in the substantia nigra and depletes dopamine in the striatum [68]. In 2021, our report identified 47 current therapies in clinical trials for PD treatment after excluding levodopa/carbidopa derivative add-on therapies [56]. We have classified these PD therapeutic strategies into 15 types: dopamine receptor agonists, anti-α-synuclein aggregation therapy, convalescent plasma therapy, cell-based therapy, gene therapy, serotonin receptor partial agonists or antagonists, monoamine reuptake inhibitors, muscarinic and nicotinic acetylcholine receptor agonists, N-methyl-D-aspartate receptor (NMDAR) modulators, anti-apoptotic drugs, kinase inhibitors, myeloperoxidase inhibitors, adenosine A_2A_ receptor antagonists, antioxidants/botanically-based medication, and others [56]. Two clinical trials have applied the nAChR agonist nicotine by transdermal patch (ClinicalTrials.gov Identifier: NCT01560754) or nasal spray (ClinicalTrials.gov Identifier: NCT03865121) in the treatment of PD, but the trial results are unclear [56]. The “cholinergic anti-inflammatory pathway” describes interactions between the CNS and the immune system via the vagus nerve, which modulates immunologic stimuli and inflammatory processes [69]. These data suggest that α7nAChR or nAChR agonists exert PD therapeutic potential, which may be associated with the cholinergic anti-inflammatory signaling pathway.

In addition to providing dopaminergic neuroprotection, α7nAChR agonists reduce levodopa-induced dyskinesias. Levodopa-induced dyskinesia (classified as peak dose dyskinesia, diphasic dyskinesia, “Off” and “On” state dystonia, and Yo-Yo-Ing) occurs in about 40% of patients after 4–6 years of levodopa therapy, greatly impairing their health-related quality of life [70,71]. MPTP-induced non-human PD primates serve as a useful animal model to study levodopa-induced dyskinesias [70,72,73]. In 1976, amantadine was approved for treating influenza virus A infection by antagonism M2 proton channel of influenza virus A. Currently, the amantadine extended-release (ER) capsule GOCOVRI™ is the only US FDA-approved treatment of levodopa-induced dyskinesias in PD patients, with or without concomitant dopaminergic medications [71]. ABT-107 is a selective α7nAChR agonist that does not affect Parkinsonism or cognitive performance; oral ABT-107 administration decreased levodopa-induced dyskinesia by 40–60% in MPTP-induced PD squirrel monkeys on levodopa/carbidopa therapy [72]. AQW051 is a selective α7nAChR partial agonist [73]. High-dose AQQ051 treatment (15 mg/kg) reduces levodopa-induced dyskinesias by 60% without compromising the benefits of levodopa and extends the duration of the levodopa antiparkinsonian response in MPTP-induced PD cynomolgus monkeys [73]. ABT-126 is an α7nAChR agonist in clinical trials for AD treatment; oral ABT-126 dose-dependently decreased dyskinesias by about 60% in MPTP-induced PD squirrel monkeys on levodopa/carbidopa therapy [74]. These results suggest that α7nAChR agonists reduce levodopa-induced dyskinesias and may have therapeutic potential as antidyskinetic agents for PD. In summary, α7nAChR may reduce the adverse effects of levodopa, including dopaminergic neuronal death and dyskinesias. Targeting α7nAChR with agonists demonstrates dopaminergic neuroprotection, which is associated with the cholinergic anti-inflammatory pathway and reduces levodopa-induced dyskinesia as a potential antidyskinetic agent in PD animal models.

## 9. α7nAChR in Schizophrenia and Therapeutic Applications

According to the most recent World Health Organization (WHO) data at the time of this review, schizophrenia affects approximately 24 million people or 1 in 300 people (0.32%) worldwide, at a rate of 1 in 222 adults (0.45%) [75]. About 0.3% to 0.7% of people are diagnosed with schizophrenia during their lifetime [76]. The prevalence of schizophrenia for males and females is similar, and the age at onset is typically in adolescence or early adulthood; onset is rare in childhood and after the fifth decade of life [77]. The hallmark of schizophrenia is psychosis and its symptoms are categorized as positive (behaviors not normally present; e.g., delusions, hallucinations, and bizarre behavior), negative (e.g., diminished emotional expression, avolition, alogia, and anhedonia), cognitive (e.g., disorganized speech, thought, and/or attention) symptoms [78]. No single schizophrenia symptom is pathogenic [78]. No diagnostic laboratory tests exist for schizophrenia; the diagnosis relies on clinical observation and self-report through an assessment of patient-specific signs and symptoms, as described in the Diagnostic and Statistical Manual of Mental Disorders, Fifth Edition (*DSM-5*) [77,78]. Multiple factors contribute to the pathophysiology and etiology of schizophrenia, including abnormalities in neurotransmission, neurochemical imbalance, genetic factors, environmental factors, and social factors [78]. Schizophrenia belongs to a group of pathologies known as complex genetic disorders, and the molecular mechanisms of schizophrenia pathophysiology remain incomplete [77]. Genetic factors constitute a crucial risk factor for schizophrenia; DNA variants (single nucleotide polymorphisms; SNPs), genetic architecture, epigenetic modification, and gene expression are thought to be associated with schizophrenia, but none are solely responsible for it. A genetic association study of 14 schizophrenia candidate genes (*RGS4*, *DISC1*, *DTNBP1*, *STX7*, *TAAR6*, *PPP3CC*, *NRG1*, *DRD2*, *HTR2A*, *DAOA*, *AKT1*, *CHRNA7*, *COMT*, and *ARVCF*) in a large European ancestry sample failed to find any significant associations between these genes and schizophrenia [79]. Thus, schizophrenia is probably multifactorial and influenced by a variety of environmental and genetic factors.

The treatment goals of schizophrenia include the targeting of symptoms, preventing relapse, and increasing adaptive functioning, so that patients can successfully integrate into their communities [78]. The treatment options for schizophrenia include pharmacologic therapy (typical and atypical antipsychotic drugs) and non-pharmacologic treatments (such as psychotherapy) [78]. Typical antipsychotics act almost exclusively on the dopamine system; atypical drugs modulate serotonin (5-HT), norepinephrine, and/or histamine neurotransmission as well [80]. Most antipsychotic drugs have proven effective in improving positive symptoms, but are limited or ineffective in dealing with negative and cognitive symptoms [81]. Clozapine is the most effective antipsychotic in terms of managing treatment-resistant schizophrenia; however, clozapine has a problematic safety profile [78]. Despite continued therapeutic advances, schizophrenia patients have two- to four-fold higher mortality rates compared with the general population, corresponding to a 15–20-year reduction in life expectancy [82]. New drugs are urgently needed that target the negative and cognitive symptoms of schizophrenia.

Preclinical and clinical data suggest that α7nAChR plays an important role in cognitive functions of schizophrenia [83]. A genome-wide linkage analysis has shown that the *CHRNA7* gene cluster maps to a region of replicated linkage on chromosome 15q13-q14 in patients with schizophrenia [84]. Inheritance of a defect in attentional disturbances in schizophrenia (a decrease in the normal inhibition of the P50 (50-millisecond latency) auditory-evoked response to the second of paired stimuli), is thought to be associated with DNA variants of the *CHRNA7* gene [84]. Other schizophrenia research has found that *CHRNA7* promoter variants at −86, −92, −143, −178, −194, and −241 bp decrease *CHRNA7* transcription; the most common variant at −86 bp decreases *CNRNA7* transcription by 20% [22]. Study evidence using [^125^I]-α-bungarotoxin to label α7nAChR expression on the cell surface has reported significantly fewer numbers of [^125^I]-α-bungarotoxin binding sites in the hippocampus in patients with schizophrenia compared with controls [28]. The study data was not related to generalized hippocampal cell loss, drug exposure at the time of death, or smoking history [28]. Moreover, lower levels of α7nAChR protein have been identified in the frontal cortex of schizophrenics compared with controls [85]. The evidence indicates that genetic variants of *CHRNA7* are associated with schizophrenia and that schizophrenic patients have fewer α7nAChRs in the hippocampus and frontal cortex compared with healthy normal individuals. Thus, α7nAChR is a relevant target for treating cognitive impairment in schizophrenia.

In two phase III clinical trials (ClinicalTrials.gov Identifier: NCT01716975 and NCT01714661) for the treatment of cognitive impairment in schizophrenia, encenicline (a selective α7nAChR partial agonist) failed to meet the co-primary endpoints for improved cognitive and clinical function [86]. A small, proof-of-concept trial reported that the partial α7nAChR agonist GTS-21 significantly improved neurocognition and P50 inhibition in schizophrenic patients [87], whereas a later phase II trial failed to show any improvement in cognition when patients with schizophrenia were treated with GTS-21 [88]. In one phase II trial, the highly selective α7nAChR full agonist (K_i_ = 1 nM) TC-5619 at doses of 1–25 mg improved cognitive and negative symptoms in 185 patients with schizophrenia receiving adjunctive quetiapine or risperidone [89], whereas in another phase II trial, TC-5619 at doses of 5 or 20 mg combined with a new-generation antipsychotic (121 patients with schizophrenia in each group) did not improve negative or cognitive symptoms [90]. AVL-3288 is a positive allosteric modulator of α7nAChR. In a phase Ib trial involving schizophrenic patients, AVL-3288 at 10 or 30 mg was well-tolerated but did not significantly affect auditory P50-evoked potential suppression or cognitive outcomes [91]. In 2021, a meta-analysis and systematic review of data from 13 randomized controlled trials in which α7nAChR agonists were added to antipsychotic treatment in patients diagnosed with schizophrenia spectrum disorder found no evidence in support of such regimens for the treatment of cognitive deficits, but there was a small effect in favor of the use of α7nAChR agonists for negative symptoms [81]. Currently available treatments for schizophrenia alleviate positive, but not cognitive or negative symptoms, so the evidence suggests that drug development targeting the α7nAChR remains a viable option for improving cognitive or negative symptoms in schizophrenic patients receiving antipsychotic drugs.

## 10. α7nAChR in Depression and Therapeutic Applications

According to the WHO, an estimated 5% of adults worldwide suffer from depression [92]. Current evidence points to a complex interaction between neurotransmitter levels and receptor regulation and sensitivity underlying the affective symptoms of depression [93]. The α7nAChR is associated with cognitive function and both presynaptic and postsynaptic α7nAChRs modulate neurotransmitter release in the brain through Ca^2+^-dependent mechanisms [7,8]. Chronic stress-induced depression is associated with an exaggerated inflammatory response in the brain; the α7nAChR regulates the cholinergic anti-inflammatory pathway by inhibiting the synthesis/release of tumor necrosis factor-α (TNF-α) and other inflammatory cytokines [94]. In a mouse model of chronic stress, chronic restraint stress altered components of central cholinergic signaling in the hippocampus, including increases in choline acetyltransferase expression and decreases in nuclear STAT3 signaling; cholinergic stimulation with GTS-21 significantly alleviated depressive-like behavior, neuroinflammation, and neuronal damage [94]. Physostigmine is a reversible acetylcholinesterase inhibitor that increases brain acetylcholine levels. In men, physostigmine and other centrally-acting cholinomimetic agents that increase central acetylcholine levels counteract mania, but may cause depression in some individuals [95]. Physostigmine in mice and rats strongly promotes depression-like behavior via central cholinergic activation [96]. In C57BL/6J mice, methyllycaconitine induced significant antidepressant-like effects in male mice but not in female mice [97]. Acetylcholine signaling via α7nAChRs in the hippocampus helps to regulate a subset of depression-like behaviors when acetylcholine is increased, such as under stressful conditions [97]. JNJ-39393406 (Janssen Pharmaceutica) is a selective positive allosteric modulator of α7nAChR undergoing development for the treatment of depressive disorders and smoking withdrawal [98]. In a phase IIa clinical trial (ClinicalTrials.gov Identifier: NCT02677207), JNJ-39393406 was administered as 100 mg/day for a week followed by 200 mg/day for a second week in 35 patients with unipolar depression, who were assessed by the Brief Assessment of Cognition in Schizophrenia (BACS) composite scores for cognition and the Montgomery-Åsperg Depression Rating Scale (MADRS) scores for depressive symptoms [98]. Although JNJ-39393406 was safe and well tolerated, there were no improvements in cognitive or depressive symptomatology [98]. The study authors speculated that the lack of efficacy could be due to the small number of patients and because any therapeutic improvements with JNJ-39393406 do not reach the threshold for clinical improvement in cognitive or depressive symptomatology [98]. Interestingly, postmortem [^125^I]-bungarotoxin autoradiographic measurements of α7nAChR expression in the hippocampal dentate gyrus, CA3, and CA1 regions and temporal cortex have indicated no significant differences between 13 patients with major depressive disorder and 15 non-depressed controls [99]. The etiology of depression and the role of α7nAChRs in depression remain unknown. More preclinical and clinical evidence is needed to determine how α7nAChRs affect depression and whether α7nAChR agonists improve this disorder.

## 11. Conclusions

The α7nAChR is the most commonly found homopentameric neuronal nAChR in the mammalian brain and is densely distributed in key brain areas, including the cortex and hippocampus. The cell-surface α7nAChR consists of five identical α7 neuronal subunits and regulates important roles in brain health and disease, such as neurotransmitter release, cognitive function, and cholinergic anti-inflammation. Genetic variations, transcriptional factors, promoter DNA methylation, tobacco smoking-induced alterations in levels of *CHRNA7* mRNA and protein expression, α7 subunit trafficking, folding, and assembly into homopentameric receptors, as well as the dysfunction of cell-surface α7nAChRs, are associated with AD, PD, schizophrenia, and depression. Drugs that act as agonists or positive allosteric modulators of α7nAChR demonstrate neuroprotection, cognitive improvement, and reduction in levodopa-induced dyskinesias in AD and PD. However, further preclinical and clinical trials are needed to determine how α7nAChR may improve cognitive deficits and negative symptoms in schizophrenia, as well as cognitive and depressive symptoms in depression.

## Figures and Tables

**Table 1:** Detailed information for the reference sequences (genomic DNA, mRNA transcripts and proteins, genomic DNA locations, protein and mRNA lengths of subunits of human neuronal nAChRs). All data are generated from the GeneCards database (https://www.genecards.org/ (accessed on 5 December 2022).

Neuronal Subunit (Protein Accession of UniProtKB)	Peptide Length (Amino Acids)	Gene (Reference Sequence of NCBI)	Gene Locus (Data from GRCh38/hg38)	DNA Strand Orientation	Cytogenetic Band by HUGO Gene Nomenclature Committee (HGNC)	mRNA Length (NCBI Reference Sequence)
α2 (Q15822)	529	*CHRNA2* (NC_000008.11, NC_060932.1)	chr8:27,459,756–27,479,883	Minus strand	8p21.2	4037-bases/NM_000742.4 3992-bases/NM_001282455.2 3987-bases/NM_001347705.2 4032-bases/NM_001347706.2 3916-bases/NM_001347707.2 3904-bases/NM_001347708.2
α3 (P32297)	505	*CHRNA3* (NC_000015.10)	chr15:78,593,052–78,621,295	Minus strand	15q25.1	3015-bases/NM_000743.5 1731-bases/NM_001166694.2
α4 (P43681)	627	*CHRNA4* (NC_000020.11)	chr20:63,343,223–63,378,401	Minus strand	20q13.33	5583-bases/NM_000744.7 5514-bases/NM_001256573.2
α5 (P30532)	468	*CHRNA5* (NC_000015.10)	chr15:78,565,520–78,595,269	Plus strand	15q25.1	3623-bases/NM_000745.4 2836-bases/NM_001307945.2 3493-bases/NM_001395171.1 2969-bases/NM_001395172.1 3091-bases/NM_001395173.1 3085-bases/NM_001395174.1 2833-bases/NM_001395175.1
α6 (Q15825)	494	*CHRNA6* (NC_000008.11)	chr8:42,752,620–42,796,392	Minus strand	8p11.21	2355-bases/NM_001199279.1 2400-bases/NM_004198.3
α7 (P36544)	502	*CHRNA7* (NC_000015.10)	chr15:31,923,438–32,173,018	Plus strand	15q13.3	6149-bases/NM_000746.6 6236-bases/NM_001190455.3
α9 (Q9UGM1)	479	*CHRNA9* (NC_000004.12)	chr4:40,335,333–40,355,217	Plus strand	4p14	2272-bases/NM_017581.4
α10 (Q9GZZ6)	450	*CHRNA10* (NC_000011.10)	chr11:3,665,587–3,671,384	Minus strand	11p15.4	2007-bases/NM_001303034.2 1940-bases/NM_001303035.2 1945-bases/NM_020402.4
β2 (P17787)	502	*CHRNB2* (NC_000001.11)	chr1:154,567,778–154,580,013	Plus strand	1q21.3	5857-bases/NM_000748.3
β3 (Q05901)	458	*CHRNB3* (NC_000008.11)	chr8:42,697,366–42,737,407	Plus strand	8p11.21	2347-bases/NM_000749.5 2480-bases/NM_001347717.2
β4 (P30926)	498	*CHRNB4* (NC_000015.10)	chr15:78,624,111–78,727,754	Minus strand	15q25.1	2596-bases/NM_000750.5 1617-bases/NM_001256567.3
Dupα7 (Q494W8)	412	*CHRFAM7A* (NC_000015.10)	chr15:30,360,566–30,393,900	Minus strand	15q13.2	3411-bases/NM_139320.2 2794-bases/NM_148911.1

## Data Availability

Not applicable.
